# Hide-and-Seek with Tiny Neotenic Beetles in One of the Hottest Biodiversity Hotspots: Towards an Understanding of the Real Diversity of Jurasaidae (Coleoptera: Elateroidea) in the Brazilian Atlantic Forest

**DOI:** 10.3390/biology10050420

**Published:** 2021-05-09

**Authors:** Gabriel Biffi, Simone Policena Rosa, Robin Kundrata

**Affiliations:** 1 Museu de Zoologia, Universidade de São Paulo, Av. Nazaré, 481, Ipiranga, São Paulo, SP 04263-000, Brazil; biffi@usp.br; 2 Instituto de Recursos Naturais, Universidade Federal de Itajubá, Av. BPS, 1303, Itajubá, MG 37500-903, Brazil; simonepolicena@unifei.edu.br; 3 Department of Zoology, Faculty of Science, Palacky University, 17. listopadu 50, 77146 Olomouc, Czech Republic

**Keywords:** Brazil, caatinga, classification, distribution, morphology, nature conservation, paedomorphism, systematics

## Abstract

**Simple Summary:**

Jurasaidae are small neotenic beetles which were only recently discovered in the Brazilian Atlantic Forest biodiversity hotspot. They have a limited dispersal propensity due to their larviform wingless females. Only adult males are capable of flight. So far, only three species classified in two genera are known. Here, we report the discovery of two new species together with a morphologically and geographically interesting population of one already described species. Our discovery is important not only for understanding the diversity of the group but also from a biogeographic point of view. For the first time, we report here the discovery of a jurasaid species from the relatively dry transitional zone between the Atlantic Forest and the Caatinga biomes. Considering our recent findings as well as the minute body size and cryptic lifestyle of Jurasaidae, we expect many more species will be discovered in the future from the Atlantic Forest and possibly also other surrounding ecoregions. Our study should motivate colleagues not only to perform field research in the eastern part of South America but also to pay special attention to yet undetermined materials deposited in local institutions, laboratories and collections.

**Abstract:**

Jurasaidae are a family of neotenic elateroid beetles which was described recently from the Brazilian Atlantic Forest biodiversity hotspot based on three species in two genera. All life stages live in the soil, including the larviform females, and only adult males are able to fly. Here, we report the discovery of two new species, *Jurasai miraculum* sp. nov. and *J. vanini* sp. nov., and a new, morphologically remarkable population of *J. digitusdei* Rosa et al., 2020. Our discovery sheds further light on the diversity and biogeography of the group. Most species of Jurasaidae are known from the rainforest remnants of the Atlantic Forest, but here for the first time we report a jurasaid species from the relatively drier Atlantic Forest/Caatinga transitional zone. Considering our recent findings, minute body size and cryptic lifestyle of all jurasaids, together with potentially high numbers of yet undescribed species of this family from the Atlantic Forest and possibly also other surrounding ecoregions, we call for both field research in potentially suitable localities as well as for a detailed investigation of a massive amount of already collected but still unprocessed materials deposited in a number of Brazilian institutes, laboratories and collections.

## 1. Introduction

Neoteny is the retention of juvenile features in fully reproductive adults due to the retardation of somatic development relative to the timing of sexual maturity [1]. In insects, such shifts in regulatory timing can result in larviform adults with wings greatly reduced or absent [2,3]. In Coleoptera (beetles), neoteny occurs mainly within the series Elateriformia; it occurs to a lesser extent in Dascilloidea [4] and much more typically in Elateroidea [5,6,7,8,9]. Soft-bodiedness, i.e., reduced sclerotization and a soft, highly flexible abdomen, is considered to be the first level of neotenic development [6]. In Elateroidea (i.e., click-beetles, fireflies, net-winged beetles, and relatives), females are usually much more affected by this phenomenon than their male counterparts, displaying various degrees of incomplete metamorphosis, from possession of a larviform abdomen only to a completely larva-like body [2,6,8]. Although previous authors considered the neoteny-related morphological modifications in Elateroidea as evidence for the monophyly of the soft-bodied groups (Cantharoidea) [10,11], molecular and morphological evidence strongly suggests that the neoteny and soft-bodiedness in Elateroidea originated multiple times independently [5,12,13].

Neotenic development can dramatically alter life histories. Neotenics have reduced ability to disperse and colonize new habitats due to the flightless females. Low-dispersing neotenics presumably follow a K-selection strategy that predominates in a stable or predictable environment. Indeed, several neotenic lineages occur exclusively in humid tropics and have limited ranges [6,8,14,15,16]. Due to their small size and cryptic lifestyle, elateroid neotenic beetles are relatively easy to overlook and hence rarely collected, and for many lineages, immature stages and presumably flightless females are unknown [8,9,13,14,17,18,19]. Interestingly, two of the four beetle families (and the only two belonging to Polyphaga) that have been erected during the 21st century based on newly collected material were neotenic elateroid lineages. These families had not been discovered earlier, most probably due to the minute, soft-bodied adult males, wingless females, cryptic lifestyle, and a presumably short lifespan [13,20,21]. The monogeneric family Iberobaeniidae, a close relative of the much more diverse Lycidae, was described based on three species from the southern part of the Iberian Peninsula in southern Europe [13,20]. Most recently, Rosa et al. [21,22] reported the discovery of Jurasaidae from the Brazilian Atlantic Forest ecoregion.

Jurasaidae were described based on 120 specimens (of which only 29 were adults) belonging to three species in two genera, i.e., *Jurasai* Rosa et al., 2020 and *Tujamita* Rosa et al., 2020. All stages usually occur in the soil just under the leaf litter, and larvae probably feed on juices of fungal hyphae. The adult females are wingless and resemble a larva in general appearance, and in both genera, they exhibit different degrees of neoteny. In *Jurasai* females, which are more affected by neoteny, only certain parts of the head are adult-like, while *Tujamita* females have an adult-like head, prothorax and legs, although still different from the corresponding parts in their male counterparts. Adult males are characterized by a soft body, abdomen with five free ventrites, apex of abdominal sternite IX with a pair of elongate lobes projecting above the genitalia, aedeagus not retracted into the abdomen, and the phallobase, sheathed by a tubular membrane, which opens anteriorly into a pair of large membranous vesicles [21]. Based on the results of molecular phylogenetic analyses, Jurasaidae were placed within the basal grade in Elateroidea, sister to the well-sclerotized rare click beetles, Cerophytidae. Although there is no obvious similarity between adults of these groups, their close relationship is supported by several larval characters including the modified mouthparts [21]. Superficially, jurasaids resemble soldier-beetles (Cantharidae) of the subfamily Malthininae, especially by the weakly sclerotized body, filiform antennae, shortened elytra (sometimes with yellowish apical spots), and the exposed aedeagus, which can be confused with the variously shaped projections of male terminalia in cantharids. However, Malthininae are a relatively rare group in the Atlantic Forest, and they can be readily distinguished from jurasaids by, e.g., the pronotum with well-defined lateral margins, fourth tarsomere bilobed, and the abdomen with eight ventrites [23].

Hitherto, all known Jurasaidae have been collected in the Atlantic Forest in southeastern Brazil. This complex biome originally consisted of a huge block of mostly evergreen to semi-deciduous forests stretching across a wide latitudinal range (ca. 5 to 30° S) of over 3000 km of the South American Atlantic coast, spanning over 1.5 million km^2^, and extending west into inland portions of Paraguay and Argentina [24,25]. Its distribution over tropical and subtropical climates across highly heterogeneous relief conditions led to exceptionally high levels of endemism and species richness [25,26]. Currently, it is home to 70% of the Brazilian human population (ca. 120 million people), holds some of the largest urban centers in South America (e.g., São Paulo and Rio de Janeiro), and also some of Brazil’s most productive land [27,28,29]. Due to urbanization, agricultural expansion, and industrialization, the Atlantic Forest experienced habitat loss and fragmentation since early European colonization. This biome currently remains in constant degradation, mostly fragmented into isolated patches of forest fragments separated by non-forested matrices. Current estimates of remaining vegetation cover of the Atlantic Forest in Brazil usually fluctuate around 9% to 16%, of which only a small fraction belong to strictly protected areas [27,30,31]. However, fragmentation and habitat loss are the greatest threats to maintaining biodiversity on the planet [32]. Therefore, the Atlantic Forest was considered one of the ‘hottest biodiversity hotspots’, which are both crucial centers of biodiversity and alarmingly imperiled by human activities [33,34].

In this study, we report the discovery of three new populations of *Jurasai*, two of which represent new species. The third one we tentatively identified as a geographically and morphologically divergent population of *J. digitusdei* Rosa et al., 2020. Our discovery is important from a biogeographic point of view since, for the first time, we report a jurasaid species from the relatively drier Atlantic Forest/Caatinga transitional zone in the Bahia state. Furthermore, our discoveries are put into the bigger picture of the entomology research and collecting activities in the Brazilian Atlantic Forest region. Considering our recent findings, minute body size and cryptic lifestyle of all jurasaids, and potentially high numbers of yet undescribed species of this family from the Atlantic Forest and possibly also surrounding ecoregions, we call for both field research in potentially suitable localities as well as for a detailed investigation of a massive amount of already collected but yet unprocessed materials deposited in a number of Brazilian institutes, laboratories and collections.

## 2. Materials and Methods

**Figure 1:**
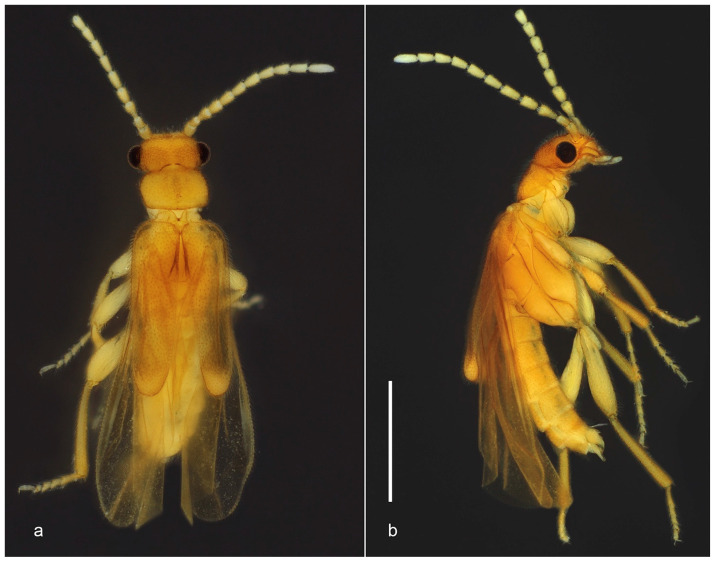
*Jurasai miraculum* sp. nov., habitus. (**a**) Dorsal view; (**b**) lateral view. Scale bar = 1.0 mm.

**Figure 2:**
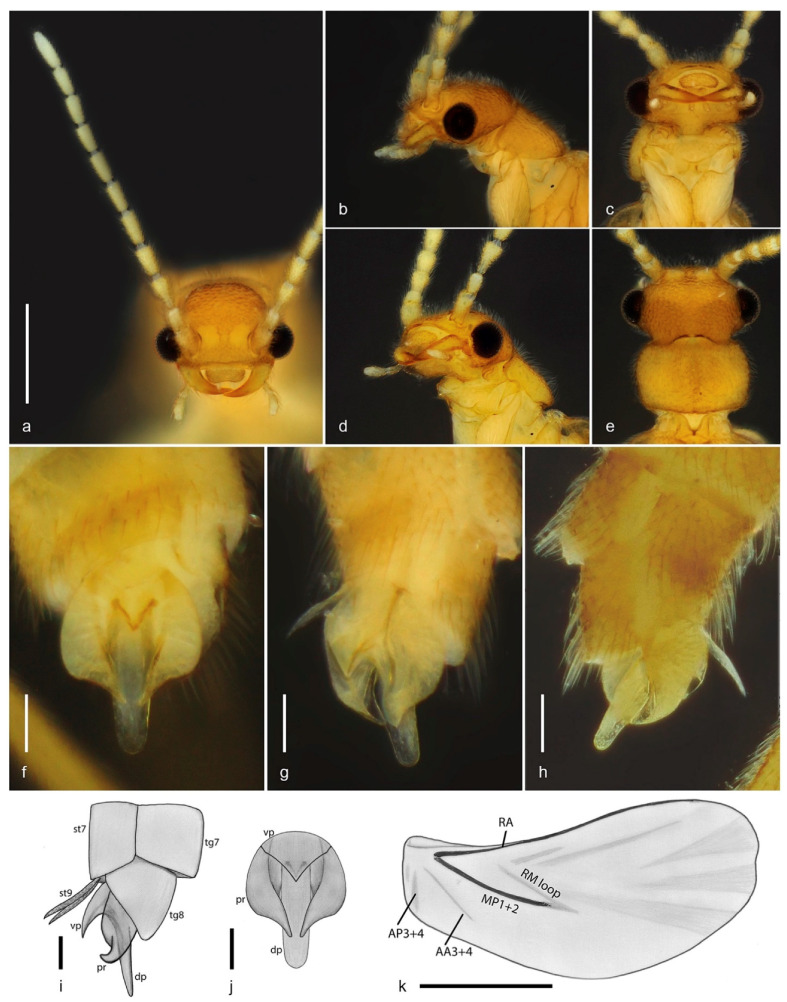
*Jurasai miraculum* sp. nov., details of morphology. (**a**) Head and antennae, anterodorsal view; (**b**) head and prothorax, lateral view; (**c**) head and prothorax, ventral view; (**d**) head and prothorax, ventrolateral view; (**e**) head and pronotum, dorsal view; (**f**–**h**) abdominal segments VII–IX, parameres and phallus: (**f**) ventral view; (**g**) ventrolateral view; (**h**) dorsolateral view; (**i**) abdominal segments VII–IX, parameres and phallus, lateral view; (**j**) parameres and phallus, ventral view; (**k**) hind wing. Abbreviations: dp: dorsal part of phallus, pr: paramere, st7: sternite VII, st9: apical lobes of sternite IX, tg7: tergite VII, tg8: tergite VIII, vp: ventral part of phallus. Scale bars: (**a**–**e**) = 0.5 mm; (**f**–**j**) = 0.1 mm; (**k**) = 1.0 mm.

**Figure 3:**
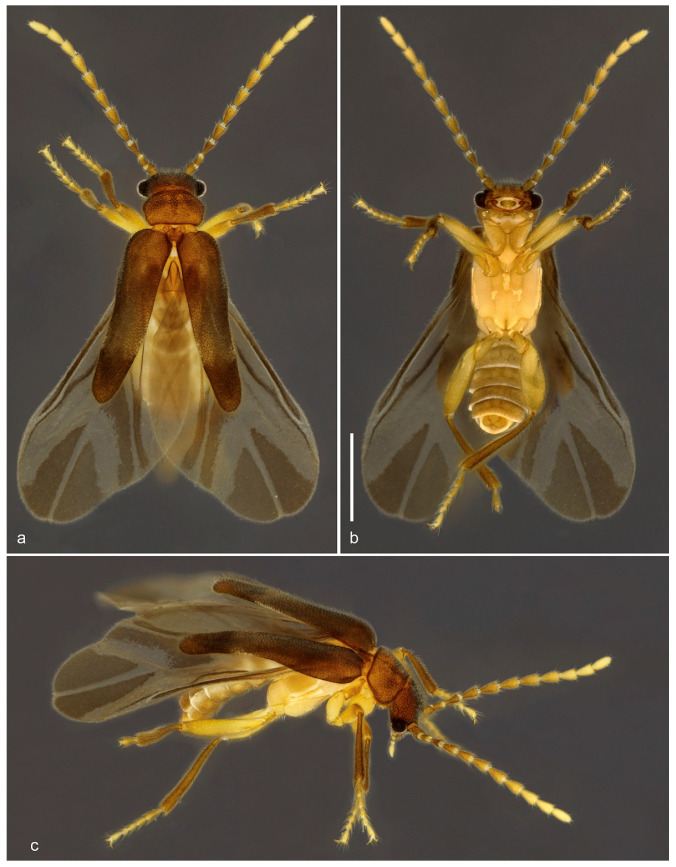
*Jurasai vanini* sp. nov., habitus. (**a**) Dorsal view; (**b**) ventral view; (**c**) lateral view. Scale bar = 1.0 mm.

**Figure 4:**
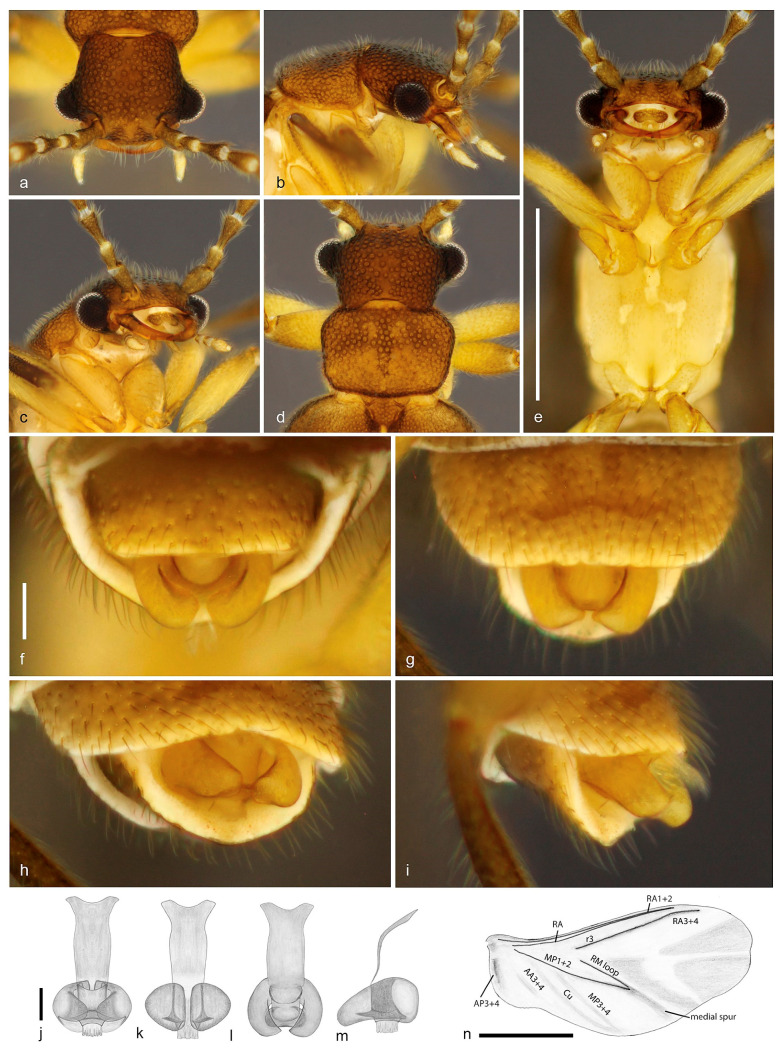
*Jurasai vanini* sp. nov., details of morphology. (**a**) Head, dorsal view; (**b**) head and prothorax, lateral view; (**c**) head and prothorax, ventrolateral view; (**d**) head and pronotum, dorsal view; (**e**) head, pro-, meso- and metathorax, ventral view; (**f**–**i**) apex of abdomen; (**f**) posteroventral view; (**g**) ventral view; (**h**) lateroventral view; (**i**) lateral view; (**j**–**m**) aedeagus; (**j**) ventral view; (**k**) dorsal view; (**l**) anteroventral view; (**m**) lateral view; (**n**) hind wing. Scale bars = 0.5 mm.

**Figure 5:**
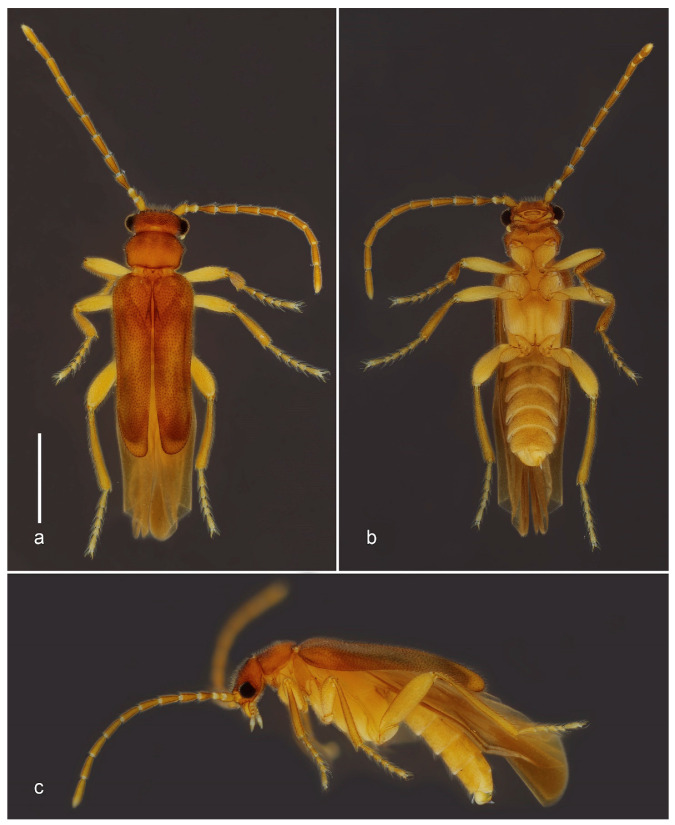
*Jurasai digitusdei* Rosa, Costa, Kramp and Kundrata, 2020, specimen from Núcleo Santa Virgínia, São Paulo state, habitus. (**a**) Dorsal view; (**b**) ventral view; (**c**) lateral view. Scale bar = 1.0 mm.

**Figure 6:**
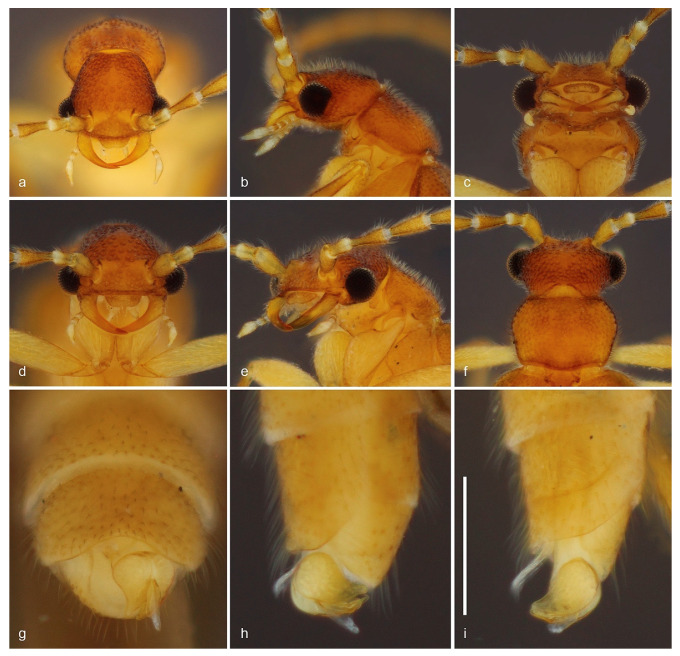
*Jurasai digitusdei* Rosa, Costa, Kramp and Kundrata, 2020, specimen from Núcleo Santa Virgínia, São Paulo state, details of morphology. (**a**) Head, anterodorsal view; (**b**) head and prothorax, lateral view; (**c**) head and prothorax, ventral view; (**d**) head, frontal view; (**e**) head and prothorax, frontolateral view; (**f**) head and pronotum, dorsal view; (**g**–**i**) abdominal segments VII–IX, parameres and phallus: (**g**) ventral view (with aedeagus approximately 160° rotated); (**h**) lateral view (with aedeagus approximately 160° rotated); (**i**) ventrolateral view. Scale bars = 0.5 mm.

**Figure 7:**
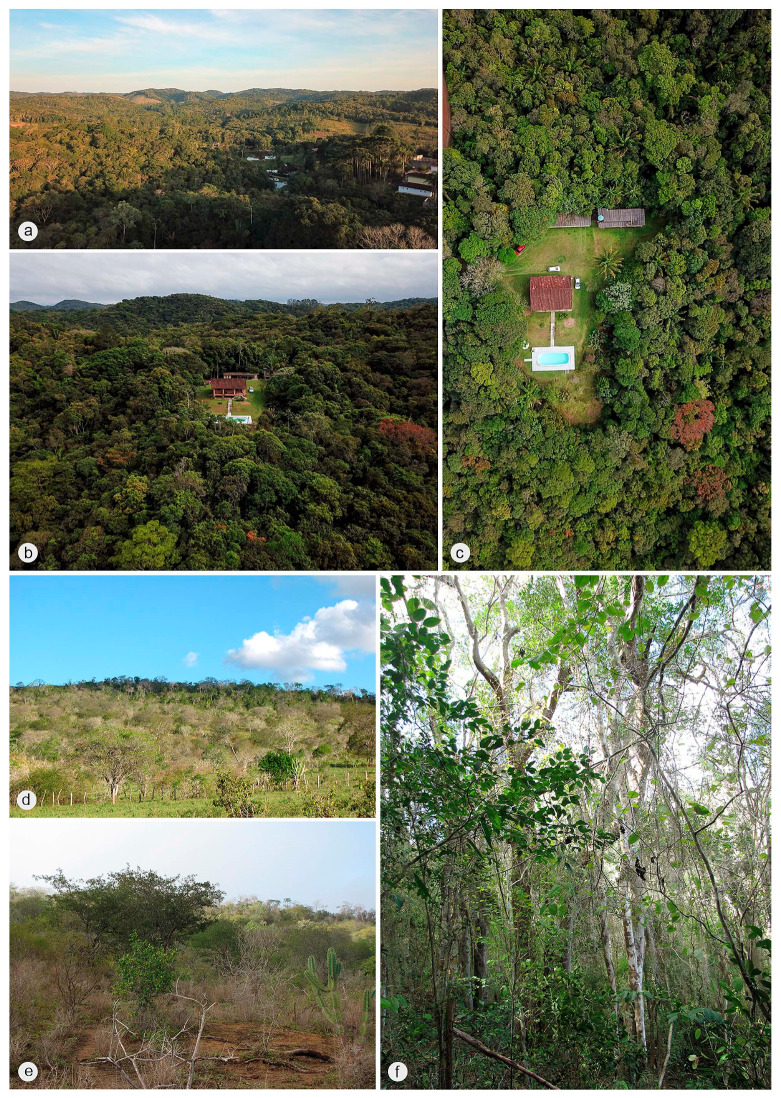
Collecting sites of *Jurasai* species (**a**–**c**) Juquitiba (São Paulo state, Brazil), type locality of *J. vanini* sp. nov., located in a region of dense ombrophilous forest; (**d**–**f**) Milagres (Bahia state, Brazil), type locality of *J. miraculum* sp. nov., located in a region of arboreal Caatinga, a seasonally dry tropical forest in a transitional area with the Atlantic Forest.

**Figure 8:**
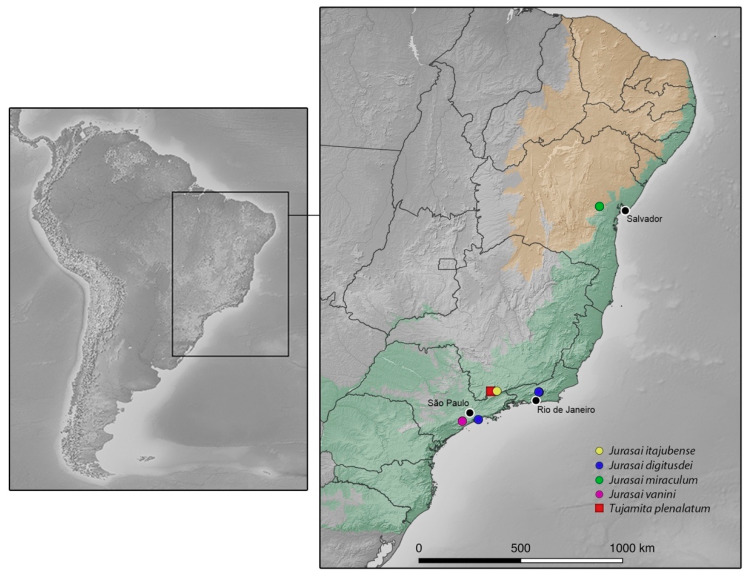
Distribution of Jurasaidae. Green hue represents the dominium of Atlantic Forest and orange hue represents the Brazilian semiarid region, including the Caatinga. Raster data available at https://www.naturalearthdata.com (accessed on 19 January 2021). Vegetation vector shapefiles available at https://www.ibge.gov.br/geociencias/downloads-geociencias.html (accessed on 19 January 2021).

A total of nine male specimens belonging to three species of genus *Jurasai* (Figure 1, Figure 2, Figure 3, Figure 4, Figure 5 and Figure 6) from three localities in eastern Brazil (i.e., Núcleo Santa Virgínia, Juquitiba and Milagres; Figure 7 and Figure 8) were studied, and they were also compared with 21 specimens of both *Jurasai* and *Tujamita* reported by Rosa et al. [21]. All newly studied specimens were collected using Malaise traps. For the exact numbers of individuals under each species, corresponding localities and information on the collecting methods, see the Results part below.

Núcleo Santa Virgínia and Juquitiba are inserted in the Serra do Mar mountain range, extending over 1000 km between Rio de Janeiro and Santa Catarina states [35], once covered by continuous Atlantic Forest. Despite being fragmented, it forms the main biological corridor from southeast to south of Brazil. The Serra do Mar within São Paulo state comprises the Serra do Mar State Park (PESM), the largest conservation unit of Atlantic forest, ranging from Atlantic plateau to coastal plain. This park benefits the biodiversity conservation and the population of 25 cities with all major categories of ecosystem services—provisioning, regulating, cultural, and supporting services. The 3320 km^2^ park is divided into eight management units, including the Núcleo Santa Virginia, a 175 km^2^ patch of mostly secondary and native ombrophilous montane forest with altitudes from 740 m to 1620 m [36]. Juquitiba is also within the Serra do Mar mountain range, but the collecting site is in a private property in the vicinity of the PESM circumscription, west of the mountain slopes of the Serra do Mar. It is characterized by dense ombrophilous vegetation, although partly anthropized, at about 730 m of altitude. Milagres is a municipality of Bahia state distant about 110 km from the coast, in a transitional area between the Caatinga and the Atlantic Forest biomes, at about 330 m of altitude. The locality is covered by arboreal caatinga, a semideciduous vegetation characterized by trees varying from 7 to 15 m in height, lower shrubs, cacti, bromeliads, and soil with a leaf litter layer [37,38].

The examined specimens have been deposited in the Museu de Zoologia da Universidade de São Paulo, São Paulo, Brazil (MZUSP) and Universidade Federal de Itajubá, Itajubá, Brazil (UNIFEI). The holotypes are preserved in alcohol. Specimens were soaked in 10% KOH for 24 h before dissection. Pronotal and elytral lengths were measured at midline, and pronotal and elytral widths at the widest part. Morphological terminology follows Rosa et al. [21]. Data from the locality labels are cited verbatim.

The line drawings were produced via camera lucida attached to a stereomicroscope. Photographs were taken on a Canon EOS Rebel T3i camera with Canon MP-E 65 mm macro-lens with additional extension tubes and StackShot macro-rail. Multi-focus images were combined with Zerene Stacker version 1.04. All photographs and illustrations were edited in Adobe Photoshop CS6. The distribution map was created with Quantum GIS version 2.18.7-1 (available at http://www.qgis.org (accessed on 19 July 2020)). The ZooBank LSID number for this publication is: urn:lsid:zoobank.org:pub:AA050277-D676-4C84-AF2C-07602D8C0B14.

## 3. Results

### 3.1. Systematics

Order Coleoptera Linnaeus, 1758

Suborder Polyphaga Emery, 1886

Series Elateriformia Crowson, 1960

Superfamily Elateroidea Leach, 1815

Family Jurasaidae Rosa, Costa, Kramp and Kundrata, 2020

Genus *Jurasai* Rosa, Costa, Kramp and Kundrata, 2020

Type species. *Jurasai itajubense* Rosa, Costa, Kramp and Kundrata, 2020; by original designation.

Diagnosis (adult male). See Rosa et al. [21]. Updated based on here-studied material as follows: body length from frons to apex of abdomen 2.5 to 3.3 mm, from frons to apex of wing 2.8 to 5.0 mm; head with antenna reaching anterior 1/3 to 2/3 of elytra; pronotum widest at anterior half or at middle, posterior half narrowed posteriad; phallus and parameres together 1.6 to 2.6 times wider than long; endophallus indistinct or emerging from dorso-apical elongate notch.

Composition. Four described species; two described by Rosa et al. [21], and other two in the present paper.

Distribution. Brazil (Bahia, Minas Gerais, Rio de Janeiro, São Paulo).

#### 3.1.1. New Species

- *Jurasai miraculum* sp. nov.

urn:lsid:zoobank.org:act:DED1F138-7241-41E5-94CD-AF3FFE336955 (Figure 1 and Figure 2).

Type material. Holotype, male, “BRASIL: Bahia, Milagres, 12°45.542′ S, 39°51.279′ W (elevation ca. 330 m), Malaise, 12–28.i.2011, caatinga arbórea, MA Ulysséa and AM Medina cols. (Coleção MZSP)” (MZUSP).

Diagnosis (based on adult male). *Jurasai miraculum* sp. nov. differs from its congeners by the following unique combination of characters: anterior margin of labrum emarginate, antennae shorter, reaching basal third of elytra, maxillary palpus four-segmented, pronotum rounded laterally, elytra shorter, covering up to abdominal segment IV (Figure 1 and Figure 2); wing with RA1 + 2 and RA3 + 4 indistinct, medial spur short, not reaching margin (Figure 2k); sternite VIII concealed, phallus 1.3 times longer than parameres, forked into ventral and dorsal parts (Figure 2f–j).

Description. Adult male. Total length from head to apex of wings 3.3 mm, up to apex of abdomen 2.8 mm. Body in dorsal view about four times longer than wide. Coloration of head, mandibles, pronotum and elytra testaceous yellow, frons with two lighter areas near antennal insertions, apex of elytra whitish; antennae, palpi, ventral pterothorax, abdomen and legs pale yellow, except apex of tibiae, slightly infuscate (Figure 1). Head in dorsal view about as long as wide, most part densely pubescent and punctate, punctures large, umbilicate; antennal insertions flat, exposed from above, separated by three times antennal insertion width; two glabrous areas between antennal insertions; posterior part to eyes 0.5 as long as anterior, narrowing posteriad; posterior margin emarginate; labrum 2.6 times wider than long, anterior margin emarginate (Figure 2a). Antenna reaching anterior third of elytra in dorsal view, densely covered with fine whitish pubescence; antennomere I cylindrical, two times longer than wide, II 1.7 times longer than wide, III longer, conical, apex twice wider than base, IV–X cylindrical, slightly shorter apicad, XI subcylindrical, narrowing at apex. Mouthparts with maxillary palpus four-segmented, palpomeres I and II subequal in length, III twice as long as II, IV 2.3 times as long as III, with apex flattened, truncate in dorsal view; anterior margin of prementum slightly sclerotized, labial palpus two-segmented, apical palpomere fusiform about five times longer than the basal one (Figure 2b–d). Pronotum 1.6 times wider than long, widest at middle, lateral margins rounded; anterior margin almost straight, posterior margin slightly rounded; anterior and posterior angles obtuse, lateral carinae absent; surface evenly densely setose-punctate, punctures umbilicate (Figure 2e); prosternal process with apex truncate; meso-metaventrite suture distinct in ventral view. Scutellar shield 1.5 times wider than long, nearly parallel-sided, posterior margin truncate, finely setose. Elytra covering abdomen up to segment IV, tapered with median edges divergent on posterior third, each elytron 3.8 times longer than wide; apex swollen; surface densely and finely punctate (Figure 1). Hind wing surpassing abdomen, 1.8 times longer than elytra (surpassing elytra by 0.8 times elytral length); RA impressed, not forked into RA1 + 2 and RA3 + 4, corresponding area of RA3 + 4 and r3 with two light linear sclerotizations; medial area with MP1 + 2 short; RM loop and medial spur weakly impressed, short, not reaching wing margin; anal notch absent, AP3 + 4 and AA3 + 4 weakly impressed; apical field with three triangular light sclerotizations occupying half of total wing length, apex weakly notched between two apicalmost sclerotizations (Figure 1 and Figure 2k). Legs with fourth tarsomere shortest, truncate at apex. Abdomen weakly sclerotized, slightly flattened dorso-ventrally, densely covered with long and fine whitish pubescence. Tergite VIII with lateral margins broadly arched ventrad, two times wider than long, evenly sclerotized and covered with long thick setae denser on posterior half; sternite VIII membranous, completely concealed underneath sternite VII; apex of sternite IX divided into two very narrow elongate lobes. Aedeagus with parameres convex laterally, lateral margins abruptly convergent on posterior third, apices tapered, convergent ventrad; phallus with ventral part short, apically triangular, dorsal part spatulate, parallel-sided, 2.5 to 3.0 times longer than ventral part and 1.3 times longer than parameres (Figure 1b and Figure 2f–j). Adult female and immature stages unknown.

Etymology. The specific epithet *miraculum* is Latin for “miracle”, in reference to the type locality called Milagres (“miracles”) in Bahia state, Brazil.

Distribution. Brazil (Bahia state).

- *Jurasai vanini* sp. nov.

urn:lsid:zoobank.org:act:070424BD-5FBF-4F0D-BBC7-5C9C36FFD187 (Figure 3 and Figure 4).

Type material. Holotype, male, “BRASIL: São Paulo, Juquitiba, Estrada dos Justos, Sítio Utopia, 23°58′19′′ S, 47°01′24′′ W (elevation ca. 730 m), Malaise, 18.x.2020–21.xi.2020, Migliore, L.; Garcia, K.; Botero, J.P. leg.” (MZUSP).

Diagnosis (based on adult male). This species differs from its congeners by the following combination of characters: anterior margin of labrum deeply emarginate (Figure 4c), antennae reaching half of elytra (Figure 3), maxillary palpus five-segmented, scutellar shield emarginate posteriorly (Figure 4d), elytra weakly tapered (Figure 3a); hindwing venation with R3, RA1 + 2, RA3 + 4, RM loop present (Figure 4n), abdominal sternite VIII concealed, parameres with longest axis perpendicular to phallus, phallus cylindrical with a subapical flap (top hat-shaped) (Figure 4f–m).

Description. Adult male. Total length from head to apex of wings 4.0 mm, up to apex of abdomen 3.2 mm. Body in dorsal view about four times longer than wide. Coloration of head, mandibles, pronotum and elytra brown, frons with two lighter areas near antennal insertions and one triangular lighter area between them, apical fourth of elytra darker, antenna with antennomeres I–III brown, gradually lighter and yellowish from IV to XI, palpi, ventral surface and legs pale yellow, except abdomen, femoral apices, and tibiae, infuscate (Figure 3). Head in dorsal view about as long as wide, densely pubescent, and punctate, punctures large, umbilicate, except on lighter areas between antennal insertions, smooth; antennal insertions slightly elevated, exposed from above, separated by 3.8 times antennal insertion width; posterior part to eyes 0.8 as long as anterior, narrowing posteriad; posterior margin emarginate; labrum 2.2 times wider than long, anterior margin deeply emarginate (Figure 4c). Antenna reaching anterior half of elytra in dorsal view, densely covered with fine brownish pubescence; antennomere I–II cylindrical, I two times longer than wide, II 1.5 times longer than wide, III–X longer, conical, apex about two times wider than base, slightly shorter apicad, XI subcylindrical, narrowing at apex (Figure 3). Mouthparts with maxillary palpus five-segmented, palpomere I two times longer than II, II–IV subequal in length, V 2.7 times as long as IV, with apex acuminate; anterior margin of prementum slightly sclerotized, labial palpus two-segmented, apical palpomere fusiform four times longer than basal one (Figure 4b,c). Pronotum 1.5 times wider than long, widest at anterior third, lateral margins sinuous; anterior margin slightly rounded, posterior margin almost straight; anterior and posterior angles obtuse, lateral carinae absent; surface densely setose-punctate, except small areas at disc, smooth; punctures umbilicate; prosternal process with apex truncate; meso-metaventrite suture distinct in ventral and lateral view (Figure 4e). Scutellar shield as long as wide, sides weakly convergent posteriad, posterior margin notched, finely setose (Figure 4d). Elytra covering abdomen up to abdominal segment V, tapered with median edges divergent on posterior two fifths, each elytron 4.3 times longer than wide; apex swollen; surface densely and finely punctate (Figure 3). Hind wing surpassing abdomen, 1.6 times longer than elytra (surpassing elytra by 0.6 times elytral length); RA1 + 2 and RA3 + 4 not contiguous, r3 elongate, MP1 + 2, RM loop distinct, medial spur blurred; corresponding areas of MP3 + 4, Cu, AA3 + 4 with light sclerotizations, AP3 + 4 distinct, anal field prominent, anal notch present; apical field with three triangular sclerotizations occupying half of total wing length; apex notched between two apicalmost sclerotizations (Figure 3 and Figure 4n). Legs with fourth tarsomere shortest, truncate at apex. Abdomen weakly sclerotized, slightly flattened dorso-ventrally, densely covered with long and fine brownish pubescence. Tergite VIII with lateral margins broadly arched ventrad, two times wider than long, evenly sclerotized and covered with long setae on posterior half; sternite VIII completely concealed underneath sternite VII, reduced to glabrous, transverse and curved sclerotized strip, 11 times wider than long; apex of sternite IX divided into two very narrow elongate lobes. Aedeagus with parameres convex laterally, somewhat conical, elongated in dorsoventral axis, which is perpendicular to phallus, apices rounded, convergent ventrad; phallus as long as parameres, cylindrical, longitudinally open between parameres dorsally, subapically marginated by a flap, apex posterior to flap very short and conical, endophallus emerging from apex; phallobase 2.6 times longer than wide, 2.3 times longer than parameres (Figure 3b and Figure 4f–m). Adult female and immature stages unknown.

Etymology. The specific epithet is patronymic, named in honor of the late Professor Sergio A. Vanin (1948 to 2020). Prof. Vanin was a major mentor for Brazilian students devoted to zoological systematics, especially of Coleoptera, the group to which he dedicated most of his career as a researcher and educator. He was overjoyed about the recent discovery of Jurasaidae.

Distribution. Brazil (São Paulo state).

#### 3.1.2. New Distributional Record and Remarks

- *Jurasai digitusdei* Rosa, Costa, Kramp and Kundrata, 2020 (Figure 5 and Figure 6).

Material examined. Seven males: 3 ex., “BRASIL, São Paulo, São Luiz do Paraitinga, PESM, Núcleo Sta. Virgínia, malaise, ponto 6, 23°19′24.8′′ S, 45°05′40.1′′ W (elevation ca. 1000 m), 21.xii.2010, N.W. Perioto and eq. col.” (MZUSP); 1 ex., “BR/SP/São Luiz do Paraitinga P.E.S.M.—Núcl. Sta. Virgínia—Malaise Ponto 3, 23°19′27.2′′ S, 45°05′38.5′′ O (elevation ca. 1000 m), 22/X/2010, N. W. Perioto e eq. cols.” (UNIFEI); 3 ex., “BR/SP/São Luiz do Paraitinga—P.E.S.M.—Núcl. Sta. Virgínia, Malaise Ponto 3 23°19′24.8′′ S, 45°05′40.1′′ W (elevation ca. 1000 m) October 22, 2010, N. W. Perioto e eq. cols.” (MZUSP).

Distribution. Brazil (Rio de Janeiro and São Paulo states).

Remarks on intraspecific variability. This species was originally described from the Parque Nacional da Serra dos Órgãos in Rio de Janeiro state. Specimens from São Paulo differ from those from Rio de Janeiro (in parenthesis) in the coloration of antennae, with antennomeres I–II yellow, III–XI light brown (antennomeres I–VII brown, antennomere VIII light brown, antennomeres IX–XI yellow) and the apical part of the elytron, which is darker or of the same color as the anterior part, with an apical margin lighter (elytral apex darker, without whitish margin); punctation of head, pronotum and elytra larger; labrum truncate to slightly emarginate (labrum truncate); elytron 4.4 to 4.6 times longer than wide (4.0 to 4.4 times longer than wide).

### 3.2. Identification Key to Genera and Species of Jurasaidae

1. Pronotum with lateral carina; elytra long, almost completely covering the hind wings, parallel-sided, median edges contiguous to apex, apices flat; tarsomere IV deeply notched*Tujamita plenalatum* Rosa et al., 2020–. Pronotum without lateral carina (Figure 2b, Figure 4b and Figure 6b); elytra short, exposing apical third of hind wing, with median edges separated and divergent apicad, lateral edges sinuate, apices swollen (Figure 1, Figure 3 and Figure 5); tarsomere IV truncate2 (*Jurasai* Rosa et al., 2020)2. Elytra strongly tapered on posterior 2/3; sternite VIII partly exposed*J. itajubense* Rosa et al., 2020–. Elytra weakly tapered on posterior 1/3 (Figure 1, Figure 3 and Figure 5); sternite VIII concealed33. Maxillary palpus four-segmented (Figure 2a,b); antenna short, reaching basal third of elytra; pronotum widest at middle (Figure 2e); phallus 1.4 times longer than parameres (Figure 2f–j)*J. miraculum* sp. nov.–. Maxillary palpus five-segmented (Figure 4b and Figure 6b); antenna long, reaching half of elytra; pronotum widest at anterior third (Figure 4d and Figure 6f); phallus as long as parameres (Figure 4j–m and Figure 6g–i)44. Labrum truncate to slightly emarginate (Figure 6a,c–e); phallus tapered (Figure 6g,h)*J. digitusdei* Rosa et al., 2020–. Labrum deeply emarginate (Figure 4c,e); phallus cylindrical with apical flap (Figure 4j–m)*J. vanini* sp. nov.

## 4. Discussion

Jurasaidae were described in 2020 based on material representing three species in two genera collected between 2015 and 2018 at two localities in the southern mountain ranges of the Atlantic Forest. While *Jurasai itajubense* and *Tujamita plenalatum* occur within the Serra da Mantiqueira mountain chain, *J. digitusdei* was described from the Serra do Mar [21]. Our recent discoveries help to significantly improve our understanding of the diversity, distribution and natural history of this enigmatic beetle family, and also emphasize several problems related to its research.

Hitherto, all jurasaid species have been known from only a limited number of specimens, and each of them has been known from a single population only. Thus, the extent of intraspecific morphological variability and distributional range size could not be addressed. Here, we report a *Jurasai* population from Núcleo Santa Virgínia, Serra do Mar State Park (PESM) in São Paulo state, which at first sight seemed to represent a new species, especially due to their generally lighter coloration. However, after a thorough morphological examination, no reliable discrete differences in the principal morphological characters, including male genitalia, were found between the new population and *J. digitusdei*. Besides coloration, differences between specimens of both populations are slight and include the shape of labrum, body punctation, and relative length of elytra. Núcleo Santa Virgínia is one of the conservation units within the PESM, 250 km distant from the type locality of *J. digitusdei*, which lies in the Parque Nacional da Serra dos Órgãos in Rio de Janeiro state [21]. Both localities belong to the Serra do Mar mountain range, which extends over 1000 km between north of Rio de Janeiro and Santa Catarina states, and was originally covered by continuous Atlantic Forest [35]. Since the specimens from Núcleo Santa Virgínia cannot be reliably distinguished from *J. digitusdei* based on adult male characters, we refrain here from erecting a new species name, and tentatively consider both populations conspecific. The discoveries of immature stages and females of both São Paulo and Rio de Janeiro populations would help us to better understand the extent of differences between these geographically distant units. Additionally, the molecular-based approaches might eventually confirm whether the specimens of these populations represent distinct species.

Both here-described species—*J. vanini* sp. nov. and *J. miraculum* sp. nov.—share the diagnostic characters of *Jurasai* [21]; on the other hand, they differ in various characters. While *J. vanini* sp. nov. is morphologically similar to both previously described *Jurasai* species, *J. miraculum* sp. nov. differs from its congeners in more characters, including the shape of pronotum (widest medially; at anterior half in remaining species), in the proportion of phallus and parameres combined (1.3 times longer than wide; 1.6 times wider than long in remaining species), and in having the endophallus not visible with its opening in the phallus indistinct (the endophallus emerges from a dorso-apical elongate notch in remaining species). What is more, *J. miraculum* sp. nov. has several characters that are unique among Jurasaidae: the frons flattened, with antennal insertions not elevated; terminal maxillary palpomere almost parallel-sided in apical half; and phallus about 1.4 times longer than parameres and forked into a short ventral part and a long dorsal part, forming a hook in lateral view. On the contrary, all other *Jurasai* species and *Tujamita plenalatum* have antennal insertions elevated, maxillary palpi apically acuminate, and phallus as long as or slightly shorter than parameres, never forked. The higher morphological similarity of *J. vanini* sp. nov. with *J. itajubense* and *J. digitusdei* on the one hand, and the morphological divergence of *J. miraculum* sp. nov. on the other hand, are in excellent agreement with the distribution of all known Jurasaidae species (Figure 8).

Jurasaidae were originally described from two localities in protected areas in Serra do Mar and Serra da Mantiqueira mountain chains, deeply nested within the southern mountain ranges of the Brazilian Atlantic Forest, where the largest and best-preserved remaining portions of this threatened biome are situated. The extremely low dispersal propensity of neotenic elateroid beetles with soil-inhabiting larvae and larviform flightless females makes them strongly dependent on long-term climatically stable habitats, including humid tropics [6,13,14,15,16]. Accordingly, one would expect that Jurasaidae are at present bound to the remnants of natural rainforest located deep within the Atlantic Forest biome and are, hence, ideal indicators of such places. Indeed, the current discoveries of a new population of *J. digitusdei* from Núcleo Santa Virgínia and *J. vanini* sp. nov. from Juquitiba support this hypothesis. Both localities lie in the Serra do Mar mountain range, which is composed of a mosaic of vegetation including lowland, sub-montane and montane dense ombrophilous forests [36]. Núcleo Santa Virgínia is a part of Serra do Mar State Park (PESM). The type locality of *J. vanini* sp. nov. is on private property in the municipality of Juquitiba, southeast of São Paulo state, in the vicinity of the PESM circumscription. The exact locality lies in an area with rural houses, with most of the natural vegetation cover preserved, though somewhat anthropized (Figure 7a–c). However, the surrounding region at Ribeira valley, partly included within the PESM, represents one of the most preserved and least explored portions of the Atlantic Forest, thus being considered one of the priority areas for conservation of biodiversity in the PESM [36].

The previous hypothesis is challenged by the first discovery of *J. miraculum* sp. nov. from the Caatinga biome in Bahia state in northeastern Brazil, located about 1100 km from where all other Jurasaidae were collected in southeastern Brazil, and almost 1000 km from the northern fringes of the Serra do Mar and Serra da Mantiqueira mountain ranges (Figure 8). Obviously, this discovery suggests that the jurasaid beetles are not endemic to the southern portions of the Atlantic Forest, and that they are not necessarily bound to dense, humid rainforests, but can survive in relatively drier and open forest habitats (Figure 7d–f). The Caatinga, often referred to as a seasonally dry tropical forest, is a broad mosaic of scrub vegetation and patches of dry forest [39,40,41,42]. Although some portions of the Caatinga are covered by low-stature vegetation which is adapted to xeric climate [39,43], most shrubs and trees consist of dry forest species rather than savanna ones [41,44]. Although the Caatinga is considered to receive less attention than any other biome in Brazil [41,45], recent studies have clearly demonstrated that this biome reaches considerably high levels of diversity and endemism for both animals and plants [39,40,46,47]. The area around the municipality of Milagres in Bahia state, the type locality of *J. miraculum* sp. nov., lies in a transitional zone between the Caatinga and the Atlantic Forest, though the vegetation and climate are typical for the Caatinga [37]. It is covered by semideciduous vegetation called “arboreal caatinga”, which is characterized by the presence of trees varying from 7 to 15 m of height, lower shrubs, cacti, bromeliads, and the soil with a leaf litter layer [39] (Figure 7d–f). That area is considered of very high priority for the conservation of the Caatinga biome [48,49]. A unique specimen was collected using a Malaise trap in January, the start of a short rainy season, when the landscape rapidly turns green [40].

The discovery of a jurasaid in the transitional area between the Atlantic Forest and the Caatinga suggests that although these beetles are probably actually more commonly found in the humid rainforest, they are also preadapted for life in somewhat drier environments. The locality where the specimen of *J. miraculum* sp. nov. was collected contains a relatively thick layer of leaf litter and relatively humid soil, which is probably the reason why these beetles can survive in this area. Members of another elateroid family containing neotenics, Lycidae, are also usually dependent on moist forest habitats and only a fraction of their diversity can be found in semi-dry regions, where they depend on rotten wood in soil [50]. Iberobaeniidae, another small-bodied neotenic beetle lineage with soil-inhabiting larvae, occupy various relatively semi-dry to dry habitats in the Mediterranean region of southern Europe [13,20]. Their relict distribution is most probably limited to sparse remnants of favorable habitats in the area. Although two species occur in the higher elevations with higher annual average precipitation, the third species is known from a very dry region. However, their dependence on water can be documented by the fact that they were predominantly collected in the early morning when relative humidity was high [20].

There are many unanswered questions regarding the historical biogeography as well as recent distribution of Jurasaidae. Since the historical distributions of both the Atlantic Forest and the Caatinga experienced many mutually exclusive expansions and contractions [42,51], it would be interesting to test whether jurasaids somewhat followed those climatically driven vegetation shifts or if they adapted to local changes and survived within a given range regardless of the climate conditions and vegetation cover. Additionally, since no immature stages or larviform females were collected in the Caatinga, further research should be carried out to find out whether the finding of a single male in that area during the beginning of the rainy season was just anecdotal and its population occupies the nearby, only 20-km-distant, more humid forest, or if there is indeed a stable population near Milagres and possibly in other relatively drier areas. However, considering that elateroid neotenics are usually not very good flyers, it is more probable that they indeed occupy also at least drier edges of the Atlantic Forest and the transitional areas with the Caatinga formation.

The surprisingly late discovery of a new beetle family in localities considered to be the best explored bits of the Atlantic Forest, although in the most endangered and fragmented region, evidently barely uncovered a cryptic hidden diversity, which needs to be better explored [21]. Our recent findings of further populations of still relatively very rare Jurasaidae in more remote and much less investigated regions suggest that the diversity of this endemic Neotropical beetle family is without any doubt much higher than previously expected. Hitherto known species were known from nearby, relatively conserved stable areas in altitudes ranging from 1100 m to 1800 m. Newly discovered populations not only significantly expanded the geographical range of the family (Figure 8) but they also showed that Jurasaidae are able to live in the partly disturbed and anthropized areas and in much lower elevations (Figure 7). All hitherto known species were collected using Malaise traps, which seem to be a very effective collecting method for flying adult males. However, due to the cryptic lifestyle of jurasaids and still very scarce data on their natural history, a combination of active collecting techniques and passive traps should be employed. The soil should be sampled for the immature stages, neotenic females and even adult males, which are often also found deeply buried in the soil [21].

Considering the here-reported discoveries which showed that Jurasaidae are distributed in a much larger area and that they can occupy a much wider variety of habitats than originally thought, we can expect that exceptionally high numbers of new species and populations will be discovered in the Atlantic Forest as well as in the surrounding drier ecosystems in near future. One would say that “more boots on the ground” [52] are needed for the field research not only in the well-preserved remnants of the rainforest in southeastern Brazil but also in the central and northern portions of the Atlantic Forest and surrounding areas, which are much less researched. Additionally, the field research on Jurasaidae (as well as other insects) may be hampered by various factors, e.g., poorly accessible localities, lack of funding and hence lack of trained people, and also by the need for official collecting permits. However, over recent decades, many research activities, including the biodiversity surveys, were carried out in the Atlantic Forest [53,54,55,56,57]. Insects were collected using a wide range of methods and traps, including those appropriate for the collection of all life stages and sexes of Jurasaidae. However, since various research groups were focused on specific goals and often on a systematically narrow spectrum of insects (e.g., ants [55], termites [54], and Hymenoptera [58]), residual undetermined materials were placed into a variety of collections, including major museums but also university museums and research laboratories. We expect that many jurasaid specimens are already being held in such collections. For example, smaller regional museums in Brazil store extraordinary but usually highly underestimated insect collections, which were built by their faculty members and students during long-term research activities, sometimes from hard-to-access and highly endangered areas. Despite their potential, university museums and local collections are in a parlous state, struggling against serious funding shortages, the lack of institutional support, the absence of trained personnel to perform technical and curatorial work, and the rising pressure for change in academic fashions, prioritizing research that attracts immediate funding [59,60,61,62].

The description of and introduction to Jurasaidae by Rosa et al. [21,22] and their subsequent dissemination to a wide audience through media had a direct impact on further research on the group since it allowed the almost immediate discovery of additional new species and populations of this group of beetles. The specimens, which were deposited in the collection of the MZUSP over many years, could finally be associated with the proper family. We strongly believe that further discoveries of Jurasaidae from various other collections (but also from remaining unsorted samples from the MZUSP) across Brazil will follow in due course. With our current study, researchers now have a second contribution to this enigmatic group of beetles in hands, which can help them to separate Jurasaidae specimens in both their freshly collected samples and previously collected materials deposited in various collections. As researchers become familiar with Jurasaidae, further new discoveries will increase our knowledge of Jurasaidae diversity, life history, evolution, relationships, and biogeography.

## 5. Conclusions

The Atlantic Forest is one of the Earth’s most endangered biodiversity hotspots, with less than 20% of its original cover remaining. Despite all of this loss, remaining forest fragments harbor exceptionally diverse fauna and flora with extremely high levels of speciation and endemism on the one hand, and the persistence of old lineages via low species extinction rates on the other hand, which makes this biome both a cradle and a museum of Neotropical diversity [31,33,63]. Many interesting insects were reported from the Atlantic Forest, including the bioluminescent larvae of Keroplatidae fungus gnats (Diptera) [64] and rove beetles (Staphylinidae) [65], the first Neotropical meropeid Mecoptera [66], ant-brood parasitizing scuttle flies (Phoridae) [67], and many others. The late discovery of Jurasaidae in the Atlantic Forest might seem surprising at first sight, but considering high numbers of recently reported new lineages in other soft-bodied elateroids, including Lampyridae [56,57,68,69,70,71,72,73,74], Lycidae [19,75], and Phengodidae [76,77], it is much less surprising. For example, Silveira et al. [57] examined the diversity of fireflies (Lampyridae) in the Serra do Órgãos (a subrange of the Serra do Mar), and, for the first time, collected 58 previously described species along with another 42 yet-undescribed species, which makes the Serra do Órgãos one of the richest firefly hotspots on Earth.

Considering the supposed antiquity of Jurasaidae, their extremely low dispersal and expanded geographic, altitudinal and habitat range as reported here, combined with the fact that neoteny and loss of flight promote speciation [15,78], we expect that many more jurasaid lineages will be found in the Atlantic Forest and surrounding drier ecosystems in the near future. If the largest reported diversity of Jurasaidae so far lies within the relatively better-explored area close to the main urbanization centers in the Atlantic Forest, how many new populations can be found in other, much less investigated, regions? How many yet-unknown species of Jurasaidae are distributed within the 1000-km gap between the localities in the southeastern mountain ranges and the type locality of *J. miraculum* sp. nov. in northeastern Brazil?

In this study, we want to motivate other scientists as well as citizen scientists to help gather as much new information as possible about Jurasaidae. This can be achieved not only via new discoveries directly in the field [52] but also by a careful examination of various institutional, museum, and laboratory collections in order to detect the jurasaids that may be hidden there, unidentified or misidentified as some other tiny soft-bodied elateroid. We believe that with public awareness of this enigmatic beetle family, our knowledge about Jurasaidae will considerably increase in the near future.

## Data Availability

Our study does not report any dataset.
